# Structural and Functional Basis for Biased Agonism at the 5-hydroxytryptamine 5A Receptor

**DOI:** 10.1186/s43556-025-00359-3

**Published:** 2025-11-12

**Authors:** Xiaoyu Zhang, Linshan Xie, Peipei Chen, Jingjing Yu, Xiaowen Tian, Lei Wang, Jiali Wei, Zhenhua Shao, Wei Yan, Zheng Xu

**Affiliations:** 1 Division of Nephrology and Kidney Research Institute, State Key Laboratory of Biotherapy, West China Hospital, Sichuan University, Chengdu, 610041 China; 2 Tianfu Jincheng Laboratory, Chengdu, 610212 China; 3 Department of Nephrology, Hainan General Hospital (Hainan Affiliated Hospital of Hainan Medical University), Haikou, China

Dear Editor,

The 5-hydroxytryptamine 5A receptor (5-HT_5A_R) belongs to the serotonin receptor family, which is classified as a group of G protein-coupled receptors (GPCRs) that are primarily associated with neuropsychiatric diseases. 5-HT_5A_R is widely expressed in the central nervous system, including the cerebral cortex, hippocampus, cerebellum, olfactory bulb and dentate gyrus. It is involved in a wild spectrum of neuropsychiatric functions, including cognition, memory, circadian rhythms, and antinociception effects [1]. 5-HT_5A_R predominantly signal through G_i/o_, and also recruit β-arrestin upon activation. Recent studies indicate that β-arrestin recruitment influence the efficacy of numerous drugs targeting serotonin receptors. Although several structures of inactive and active 5-HT_5A_R have been resolved, revealing interactions with several ligands as well as G_i/o_ proteins [2, 3], the role of 5-HT_5A_R-targeting drugs in β-arrestin signaling remains poorly understood. The synthetic agonist 5-carboxamidotryptamine (5-CT) is commonly used as a tool ligand to evaluate the function of serotonin receptors, particularly the 5-HT_5A_R, given its ten-fold higher affinity compared to 5-HT [4]. While 5-HT induces diverse signaling profiles across many other serotonin receptors, the biased agonism of 5-HT and 5-CT acting on 5-HT_5A_R remain unclear.

To understand the molecular mechanism of 5-HT_5A_R signaling through G_i_ and β-arrestin, we analyzed the structure of 5-CT stimulates 5-HT_5A_R/G_i1_ complex by cryo-electron microscopy (EM) with an overall resolution of 3.13 Å (Fig. S1a-b). The residues of 5-HT_5A_R and ligand were well defined in the density map, except for the intracellular loop 3 (ICL3) due to the high flexibility (Fig. 1a). By constructing the receptor residue-ligand atom contact matrix, we found 5-CT form tight hydrophobic interactions with C125^3.36^ and F301^6.51^ and salt bridge with D121^3.32^ (Fig. 1b). The amide group and the indole amine of 5-CT also interact with S204^5.42^ and T126^3.36^ by hydrogen bond (Fig. 1b). The binding pose of 5-CT exhibits slight conformational differences compared to that in a previous determined 5-CT/5-HT_5A_R/G_i_ complex structure, indicating heterogeneity of 5-CT/5-HT_5A_R interaction. This heterogeneity aligns with observations across multiple GPCR structures and likely captures near-native dynamics inherent to pharmacological mechanism of receptor-drugs interactions.

**Fig. 1:**
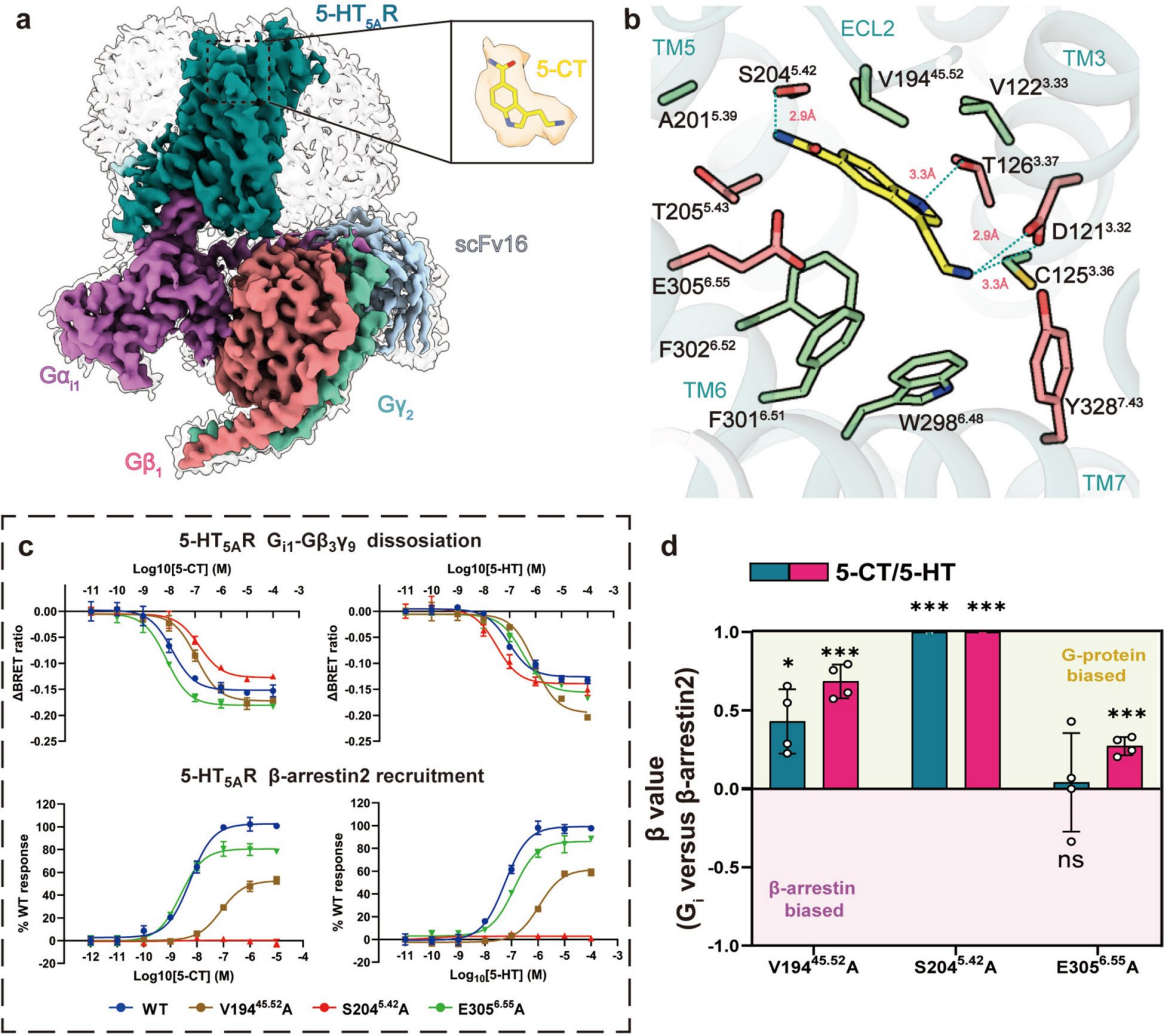
The structure and signaling characterization of 5-CT binding to 5-HT_5A_R. **a** Cryo-EM density map and atomic model of the 5-CT/5-HT_5A_R/G_i1_/scFv16 complex. (5-HT_5A_R: dark cyan; Gα_i1_: orchid; Gβ_1_: light coral; Gγ_2_: medium aquamarine; 5-CT: yellow). **b** The binding mode of 5-CT in the binding pocket of 5-HT_5A_R, the side chains of residues are shown as stick. **c** The effects of 5-CT and 5-HT on different mutants of the 5HT_5A_R. The response curves for 5-CT induced G_i1_ signaling (upper) and β-arrestin2 recruitment (lower) were measured by BRET and NanoBiT, respectively. **d** Bias factors (β value) of mutations relative to wild type (WT) calculated among three-agonist-induced receptor activation. Bias factors were derived from the curve fit parameters. Data are presented as mean ± SEM from four independent experiments, performed in triplicate. ∗ *p* < 0.033, ∗  ∗ *p* < 0.002 and ∗  ∗  ∗ *p* < 0.001 by one-way ANOVA followed by Dunnett’s multiple comparisons test.

In the contact matrix, S204^5.42^ and E305^6.55^ exhibit the highest contact frequencies with 5-CT (data not shown). To investigate the roles of these residues in 5-CT recognition, we introduced alanine substitutions at S204^5.42^ and E305^6.55^ into the receptor, following characterization of G_i1_ activation by bioluminescence resonance energy transfer (BRET) and β-arrestin2 recruitment by NanoBiT. S204^5.42^A abolished the β-arrestin2 recruitment and retained efficacy in G_i1_ dissociation (Fig. 1c), indicating that the polarity of 5.42 was essential for β-arrestin2 recruitment. Additionally, E305^6.55^A slightly enhanced the efficacy in G_i1_ signaling (Δ*E*_max_ = 0.229 ± 0.07, *p* = 0.03) and potency in the β-arrestin2 recruitment (ΔpEC_50_ = 0.452 ± 0.08, *p* = 0.006) of 5-CT. These results indicated that S204^5.42^ are essential for signaling bias of 5-CT stimulating 5-HT_5A_R, and interaction with E305^6.55^ may not be essential for 5-CT to function. Although V194^45.52^ did not form intense interaction with 5-CT, V194^45.52^A reduced potency for both G_i_ dissociation (ΔpEC_50_ = -0.841 ± 0.1, *p* < 0.001) and β-arrestin2 recruitment (ΔpEC_50_ = -1.151 ± 0.08, *p* < 0.001), indicating its importance in ligand binding. Given its strategic location on ECL2, this residue is hypothesized to function as a putative gatekeeper for the binding pocket. The loss of its bulky side chain is expected to accommodate easier ligand dissociation, thereby preventing sustained receptor activation.

We also characterized signaling of 5-HT_5A_R mutants activated by endogenous ligand 5-HT as reference. S204^5.42^A decreased potency of 5-CT (ΔpEC_50_ = -0.952 ± 0.12, *p* < 0.001), but enhanced potency of 5-HT in G_i_ dissociation (ΔpEC_50_ = 0.694 ± 0.05, *p* < 0.001). V194^45.52^A exhibited similar impacts on 5-CT and 5-HT, reducing potency of 5-CT (G_i_: ΔpEC_50_ = -0.841 ± 0.1, *p* < 0.001; β-arrestin2: ΔpEC_50_ = -0.952 ± 0.12, *p* < 0.001) and 5-HT (G_i_: ΔpEC_50_ = -1.151 ± 0.08, *p* < 0.001; β-arrestin2: ΔpEC_50_ = -1.206 ± 0.03, *p* < 0.001) in both pathways, whereas E305^6.55^A differently altered signaling of the two ligands (Fig. 1c). Specifically, E305^6.55^A slightly enhanced potency of 5-CT in β-arrestin recruitment (ΔpEC_50_ = 0.452 ± 0.08, *p* = 0.006), while it reduced potency of 5-HT in β-arrestin recruitment (ΔpEC_50_ = -0.262 ± 0.04, *p* = 0.01) thereby biasing signaling of 5-HT towards G_i_ dissociation (Fig. 1d). These findings confirmed that S204^5.42^ plays a crucial role in β-arrestin2 signaling by 5-HT_5A_R. In contrast, E305^6.55^ exhibits differential interaction modes with hydroxyl group of 5-HT compared to amide group of 5-CT. Mutagenesis and molecular dynamics simulations reveal distinct functional roles: D121^3.32^ (Energy = -41.5 ± 9.5 kcal/mol, 55.4% of total) and E305^6.55^ (Energy = -17.7 ± 10.8 kcal/mol, 23.6% of total) dominate 5-CT binding energetics, whereas S204^5.42^ facilitates β-arrestin recruitment through conformational rearrangement without significant binding energy contributions (Fig. S1c). Most serotonin receptors do not possess a negatively charged residue at position 6.55. Notably, the exceptions, 5-HT_1E_R and 5-HT_1F_R, exhibit low affinity for 5-CT. This correlation suggests that the presence of an acidic acid at 6.55 (E^6.55^) may negatively regulate 5-CT binding. Interestingly, in 5-HT_5A_R simulation, E305^6.55^ contributes a substantial favorable portion to the binding energy of 5-CT. We noticed that residues at 6.55 and 45.52 occupy roughly opposing positions. In 5-HT_1E_R and 5-HT_1F_R the amide-binding cavity is already constricted by the bulky I^45.52^, and the presence of E^6.55^ introduces steric clash. In contrast, V194^45.52^ in 5-HT_5A_R widens this cavity, allowing E305^6.55^ to form a productive hydrogen bond with the 5-carboxamide group of 5-CT, thereby playing a positive, selectivity-determining role.

Together, our results demonstrated that S204^5.42^A differentially affected the activation of downstream signaling by 5-CT and 5-HT, showing a clear β-arrestin2 signaling bias. The residue at 5.42 has been discovered contributing into the β-arrestin biased pharmacologic action of ligands in the serotonin receptors, as literatures reported [5]. The diversity of the molecular mechanism between serotonin receptors raised the possibility of designing selectively biased ligand for specific receptor in serotonin receptor family.

## Supplementary Information

Supplementary Material 1.

## Data Availability

Cryo-EM coordinates and density maps of 5-CT/5-HT_5A_R/G_i1_/scFv16 complex have been deposited at the PDB and the Electron Microscopy Data Bank under accession codes 9W6W, EMD-65707. Any additional data reported in this paper is available from the lead contact upon request.
