# Supplementary material for: Structural and Functional Basis for Biased Agonism at the 5-hydroxytryptamine 5A Receptor
Source: Mol Biomed. 2025 Nov 12;6:107. doi: 10.1186/s43556-025-00359-3 (PMC12612430; doi:10.1186/s43556-025-00359-3)
Supplement: Supplementary file 1 — Supplementary Material 1. [file 43556_2025_359_MOESM1_ESM.pdf]

Structural and Functional Basis for Biased Agonism at the 5-hydroxytryptamine 5A Receptor

Xiaoyu Zhang[1,2,#] , Linshan Xie[1,#], Peipei Chen[1,#], Jingjing Yu[1], Xiaowen Tian[1], Lei Wang[1], Jiali Wei[3,*], Zhenhua Shao[1,2,*], Wei Yan[1,*], Zheng Xu[1,*]

[1]Division of Nephrology and Kidney Research Institute, State Key Laboratory of Biotherapy, West China Hospital, Sichuan University, Chengdu 610041, China.
[2]Tianfu Jincheng Laboratory, Chengdu 610212, China.
[3]Department of Nephrology, Hainan General Hospital (Hainan affiliated hospital of Hainan medical university), Haikou, China.

[#]These authors contributed equally.

*Correspondence: weijaili@163.com (J.W.); zhenhuashao@scu.edu.cn (Z.S.); weiyan2018@scu.edu.cn (W.Y.); zhengxu@scu.edu.cn (Z.X.).

Supplementary Information

Methods

Constructs

The human $5HT_{5A}R$ fragment with 8 residues truncated at N terminus was cloned into the pFastBac1 (Invitrogen) vector and expressed in sf9 insect cell. For purification and expression, a hemagglutinin (HA) signal sequence connected to a Flag epitope tag followed by the thermostabilized apocytochrome b562RIL (BRIL) was fused to the N-terminus after the truncated $5HT_{5A}R$. Human dominant-negative (DN) $G\alpha_{i1}$ (S47N, G203A, E245A, A326S) was cloned into the pFastBac1 vector, $G\beta_1$ with N-terminal 6×His-tag and bovine $G\gamma_2$ were cloned into a pFastBac-dual vector for co-expression.

Expression and purification of 5-CT/5-$HT_{5A}R$/G_{i1}/scFv16 complex

5-$HT_{5A}R$, $G\alpha_{i1}$ and $G\beta_1\gamma_2$ constructs were co-infected at a ratio of 1:1:1 in sf9 insect cells. After 48 h, the cells were collected and lysed in buffer containing 20 mM HEPES (pH 7.5), 50 mM NaCl, 2 mM $MgCl_2$, 2 mM $CaCl_2$, 160 μg/mL benzamidine, 100 μg/mL leupeptin, 12 mU/ml apyrase, and 10 μM 5-CT for 2 h. Then added 20 mM HEPES (pH 7.5), 100 mM NaCl, 2 mM $MgCl_2$, 2 mM $CaCl_2$, 0.5% lauryl maltose neopentyl glycol (LMNG; Anatrace) (w/v), 0.1% cholesteryl (w/v), 10% glycerin (w/v), 160 μg/mL benzamidine, 100 μg/mL leupeptin, 10 μM 5-CT, 7.2 mg/mL scFv16 (expressed as previous reported[1]), and 25 mU/mL apyrase incubated for 2 h at 4 °C. The mixture was centrifuged at 85,000 × g for 30 min and the supernatant was incubated by anti-FLAG antibody for 2 h at 4 °C. The resin was washed of wash buffer containing 20 mM HEPES (pH 7.5), 100 mM NaCl, 2 mM $MgCl_2$, 2 mM $CaCl_2$, 5 μM 5-CT, 0.01% (w/v) LMNG and 0.001% (w/v) cholesteryl hemisuccinate (CHS). The protein was eluted with 5 mM EDTA and 0.2 mg/mL FLAG peptide and loaded onto a Superose6 increase 10/300 column (GE Healthcare) with buffer containing 20 mM HEPES (pH 7.5), 100 mM NaCl, 5 mM $MgCl_2$, 0.00075% (w/v) LMNG, 0.00025% glyco-diosgenin (GDN; Anatrace) and 0.0002% (w/v) CHS, and 5 μM 5-CT.

Cryo-EM data acquisition and structure resolving

Cryo-EM samples were prepared as previously described[1]. 5-CT/5-$HT_{5A}R$/G_{i1}/scFv16 complex was transferred onto a glow-discharged holey carbon grid (Quantifoil R1.2-1.3) before blotted and plunge-frozen into liquid ethane using the FEI Vitrobot Mark IV. Data were collected using a FEI Titan Krios microscope at 300 kV, equipped with the K3 Summit direct electron detector (Gatan) in super-resolution mode at a nominal magnification of ×130,000, corresponding to pixel size of 0.92Å. A total of 3,118 movies were acquired with the defocus values ranging from -1.0um to -1.8um, each consisting of 32 frames and an electron dose of 57.6 $e^-/Å^2$. Movies were motion-corrected and a total of 7,094,461 particles were picked. 2,015,134 particles were selected after three rounds of 2D classification. 314,278 particles were conserved for non-uniform refinement and local refinement after two rounds of ab-initio reconstruction and heterogenous refinement, generating a final map at a resolution of 3.13 Å (gold-standard Fourier shell correlation, 0.143). The structure was built in Coot[3] based on a previous 5-$HT_{5A}R$ complex structure and further refined using PHENIX[4].

Functional assays

BRET assay was used to detect $G\alpha_{i1}$ dissociation as previously described[1]. HEK293 cells

were co-transfected with wild type (WT) 5-HT_{5A}R or mutants, Gα_{i1}-NanoLuc luciferase (Nluc), Gβ_3 and Gγ_9-mVenus plasmid 5 µM coelenterazine h (Nanolight, CAT# 301) and ligand in HBSS were added to cells before BRET ratio (535 nm/460 nm) measurement. With the Synergy H1 microplate reader (TECAN).

The based-NanoBiT β-arrestin2 recruitment assay as previously described[1]. The recombinant plasmids 5-HT_{5A}R-LgBiT and SmBiT-β-arrestin2 were co-transfected HBSS, 5 µM coelenterazine h (Nanolight, CAT# 301) and ligand in HBSS were added to cells before luminescence (460-480 nm) measurement using the Synergy H1 microplate reader (BioTek). The ligand-induced signal was defined as fold-change to the baseline luminescence. The data statistical fitting of data such as recruitment capacity, EC_{50}, and E_{max} values was performed in GraphPad Prism 9.5.

Calculation of β value

The bias factors (β value) were determined by applying the following equation[1]:

$$\beta\ \text{value} = log_{10}\left(\left[\frac{E_{\max,P1}}{EC_{50,P1}} \times \frac{EC_{50,P2}}{E_{\max,P2}}\right]_{mutant} \times \left[\frac{E_{\max,P2}}{EC_{50,P2}} \times \frac{EC_{50,P1}}{E_{\max,P1}}\right]_{WT}\right)$$

where P1 is NanoBiT data; P2 is BRET data; β value >0 denotes G_i-protein biased and β value <0 is β-arrestin biased. Parameters used in this equation were based on the curve fits of the combined datasets described above.

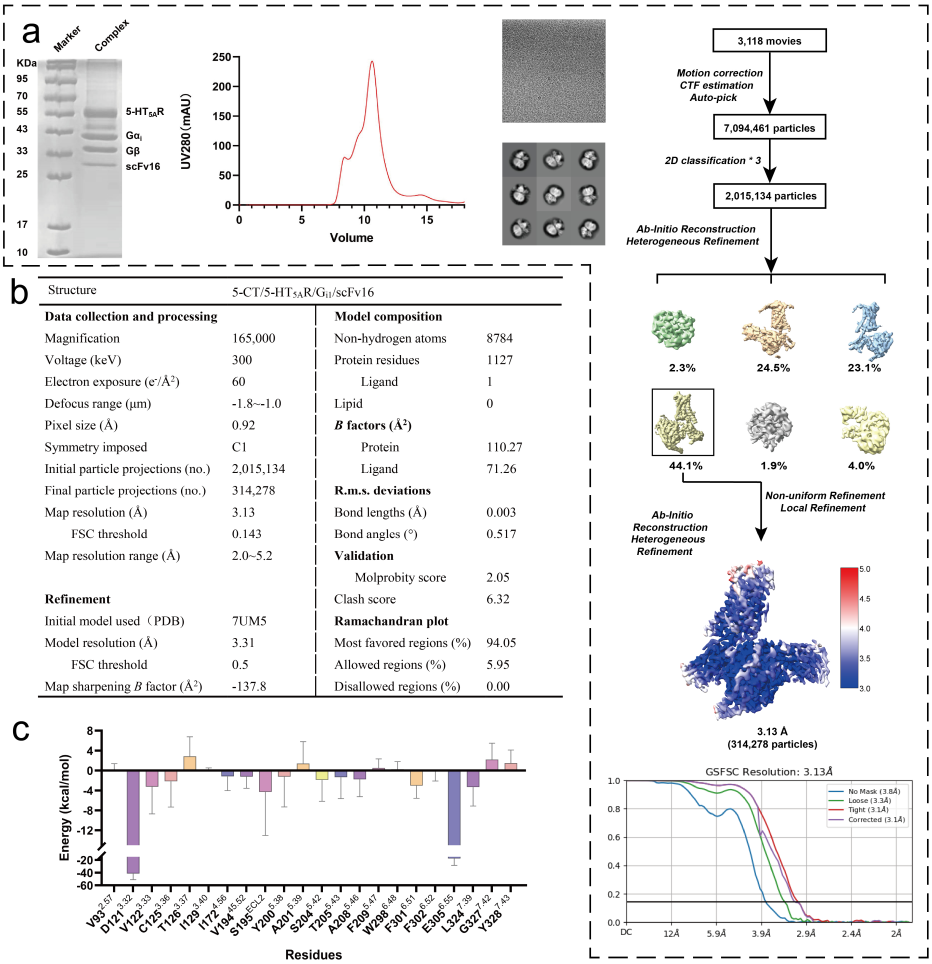

Structure	5-CT/5-HT_{5A}R/G_{i1}/scFv16		
**Data collection and processing**		**Model composition**	
Magnification	165,000	Non-hydrogen atoms	8784
Voltage (keV)	300	Protein residues	1127
Electron exposure (e⁻/Å²)	60	Ligand	1
Defocus range (μm)	-1.8~-1.0	Lipid	0
Pixel size (Å)	0.92	***B* factors (Å²)**	
Symmetry imposed	C1	Protein	110.27
Initial particle projections (no.)	2,015,134	Ligand	71.26
Final particle projections (no.)	314,278	**R.m.s. deviations**	
Map resolution (Å)	3.13	Bond lengths (Å)	0.003
FSC threshold	0.143	Bond angles (°)	0.517
Map resolution range (Å)	2.0~5.2	**Validation**	
		Molprobity score	2.05
**Refinement**		Clash score	6.32
Initial model used（PDB)	7UM5	**Ramachandran plot**	
Model resolution (Å)	3.31	Most favored regions (%)	94.05
FSC threshold	0.5	Allowed regions (%)	5.95
Map sharpening *B* factor (Å²)	-137.8	Disallowed regions (%)	0.00

Supplementary Fig. 1: a. SDS-PAGE analysis and analytical size-exclusion chromatography of the purified complex, and the cryo-EM data processing and density map of the 5-CT/5-HT_{5A}R/G_{i1}

complex. **b.** The detailed information of the cryo-EM data collection, refinement and validation statistics. **c.** Binding energy contribution of each residue within 5 Å of 5-CT in complex structure. Mean of energy contribution in final 100 ns out of total 500 ns simulation were calculated. Data represented as mean ± SD.

References

1. Feng Y, Zhao C, Deng Y, Wang H, Ma L, Liu S, et al. Mechanism of activation and biased signaling in complement receptor C5aR1. Cell Research. 2023;33(4):312–24. https://doi.org/10.1038/s41422-023-00779-2.
2. Mastronarde DN. SerialEM: A Program for Automated Tilt Series Acquisition on Tecnai Microscopes Using Prediction of Specimen Position. Microscopy and Microanalysis. 2003;9(S02):1182–3. https://doi.org/10.1017/s1431927603445911.
3. Emsley P, Cowtan K. Coot: model-building tools for molecular graphics. Acta Crystallogr D Biol Crystallogr. 2004;60(Pt 12 Pt 1):2126–32. https://doi.org/10.1107/s0907444904019158.
4. Adams PD, Afonine PV, Bunkóczi G, Chen VB, Davis IW, Echols N, et al. PHENIX: a comprehensive Python-based system for macromolecular structure solution. Acta Crystallogr D Biol Crystallogr. 2010;66(Pt 2):213–21. https://doi.org/10.1107/s0907444909052925.
